# Neurochemical and electrical modulation of the locus coeruleus: contribution to CO_2_drive to breathe

**DOI:** 10.3389/fphys.2014.00288

**Published:** 2014-08-05

**Authors:** Débora de Carvalho, Luis G. A. Patrone, Camila L. Taxini, Vivian Biancardi, Mariane C. Vicente, Luciane H. Gargaglioni

**Affiliations:** ^1^Department of Animal Morphology and Physiology, Faculty of Agricultural and Veterinarian Sciences, Universidade Estadual Paulista – São Paulo State UniversityJaboticabal, Brazil

**Keywords:** rodent, chemosensitive signaling, serotonin, glutamate, hypercapnia, ATP

## Abstract

The locus coeruleus (LC) is a dorsal pontine region, situated bilaterally on the floor of the fourth ventricle. It is considered to be the major source of noradrenergic innervation in the brain. These neurons are highly sensitive to CO_2_/pH, and chemical lesions of LC neurons largely attenuate the hypercapnic ventilatory response in unanesthetized adult rats. Developmental dysfunctions in these neurons are linked to pathological conditions such as Rett and sudden infant death syndromes, which can impair the control of the cardio-respiratory system. LC is densely innervated by fibers that contain glutamate, serotonin, and adenosine triphosphate, and these neurotransmitters strongly affect LC activity, including central chemoreflexes. Aside from neurochemical modulation, LC neurons are also strongly electrically coupled, specifically through gap junctions, which play a role in the CO_2_ ventilatory response. This article reviews the available data on the role of chemical and electrical neuromodulation of the LC in the control of ventilation.

## INTRODUCTION

Locus coeruleus (LC) is pontine region situated bilaterally on the floor of the fourth ventricle, and produces the majority of noradrenaline (NA) in the forebrain. The LC region is a densely packed nucleus, comprising about 1500 neurons per side (Aston-Jones et al., 1984). The first description of the LC was published by Reil (1809), but the term LC was proposed by the anatomists Wenzel and Wenzel in 1812 (reviewed by Russell, 1955). In addition to NA, the cell bodies also secret several neuropeptides (Sutin and Jacobowitz, 1991), among which are vasopressin, somatostatin, neuropeptide Y, enkephalin, neurotensin, CRH, and galanin (for review, see Olpe and Steinmann, 1991). This nucleus has been described in a wide variety of animals, such as frogs, birds, rodents, and primates (González and Smeets, 1993).

LC NA neurons projects broadly throughout the neuraxis, from spinal cord to neocortex (Swanson and Hartman, 1975; Foote et al., 1983; Berridge and Waterhouse, 2003). In fact, it is estimated that ∼50% of all the noradrenergic projections in the central nervous system (CNS) originate in the LC which are directed toward the forebrain, cerebellum, brainstem and spinal cord (Aston-Jones et al., 1995; Berridge and Waterhouse, 2003). LC is the primary source of an extensive, yet regionally specialized, noradrenergic innervation of the forebrain (Berridge and Waterhouse, 2003) and it is considered a major wakefulness promoting nucleus with activation of the LC resulting in an increase in EEG signs of alertness (Samuels and Szabadi, 2008).

The alerting effect of LC activation results from dense excitatory projections to the cerebral cortex, dorsal raphe, pedunculopontine tegmental nucleus and the laterodorsal tegmental nucleus, and from substantial inhibitory projections to sleep-promoting GABAergic neurons of the basal forebrain and the ventrolateral preoptic area (Samuels and Szabadi, 2008). Additionally, LC also modulates autonomic function through direct output to sympathetic and parasympathetic preganglionic neurons of the intermediolateral cell column of the spinal cord and to projections innervating other autonomic nuclei (Nygren and Olson, 1977; Westlund et al., 1983; Jones and Yang, 1985), among which known examples are the Edinger-Westphal Nucleus (Breen et al., 1983), paraventricular nucleus (Swanson and Sawchenko, 1983; Jones and Yang, 1985), caudal raphe (Hermann et al., 1997), and rostroventrolateral medulla (Van Bockstaele et al., 1989).

LC is innervated by fibers that contain many neurotransmitters such as opiates, glutamate, gamma-aminobutyric acid (GABA), serotonin, epinephrine, and the peptide orexin/hypocretin (Aston-Jones et al., 1995). The nucleus paragigantocellularis lateralis (PGi) is a source for glutamate, GABA, enkephalin, corticotrophin releasing hormone (CRH), and epinephrine. A strongly inhibitory GABA and enkephalin input originates from the dorsomedial rostral medulla, whereas orexin/hypocretin inputs originate in the hypothalamus (Horvath et al., 1999), as do histaminergic inputs (Pollard et al., 1978).

Because of these widespread projections, LC is implicated in the control of many physiological functions, including control of respiration (Oyamada et al., 1998; Fabris et al., 1999; Hilaire et al., 2004; Putnam et al., 2004; Viemari et al., 2004; Biancardi et al., 2008; de Carvalho et al., 2010; de Souza Moreno et al., 2010; Gargaglioni et al., 2010; Biancardi et al., 2014; Patrone et al., 2014) and cardiovascular function (Sved and Felsten, 1987; Yao and Lawrence, 2005; Biancardi et al., 2014; Patrone et al., 2014). LC is considered a central CO_2_/pH chemoreceptor site in amphibians and mammals (Noronha-de-Souza et al., 2006; Gargaglioni et al., 2010; Santin and Hartzler, 2013) and more than 80% of its neurons are chemosensitive, responding to hypercapnia with an increased firing rate (Pineda and Aghajanian, 1997; Oyamada et al., 1998; Filosa et al., 2002). Local acidification of noradrenergic LC neurons increases respiratory frequency and phrenic nerve discharge in cats (Coates et al., 1993). Lesion of approximately 80% of noradrenergic neurons of LC by using 6-OHDA elicited a large decrease in the response to CO_2_, of approximately 64%, due to a decreased tidal volume (V_T_), indicating that this nucleus exerts a profound effect on the hypercapnic ventilatory response (Biancardi et al., 2008). The objective of this review is to summarize the available data on the role of chemical and electrical neurotransmission in the LC in the control of ventilation during CO_2_ challenge.

## ELECTRICAL MODULATION OF THE LC

The traditional concept of cell communication is based on the presumption that the electrical coupling was a “primitive” form of signaling commonly present in invertebrates, since the chemical synapses are characterized as the predominant form of intercellular signaling in the CNS of vertebrates (Naus and Bani-Yaghoub, 1998). However, studies have shown that electrical coupling appears to be an important signaling mechanism for several functions, including cardiorespiratory regulation. In this regard, electrical communication is present in regions of the CNS involved in the generation and modulation of respiratory rhythm, inspiratory motoneuron synchronization, central chemoreception and cardiovascular control (Davidson and Baumgarten, 1988; Nattie, 1999; Solomon et al., 2001; Solomon, 2003; Rash et al., 2007; Pierce et al., 2010). In fact, the presence of gap junctions has been reported in many regions that are considered CO_2_/pH chemosensitive such as the caudal medullary raphe, retrotrapezoid nucleus (RTN), dorsal motor nucleus of the vagus (DMV), nucleus of the solitary tract (NTS), and the LC, thus implying that electrical coupling may constitute an important mechanism for CO_2_ drive to breathe (Dean et al., 1997; Rekling and Feldman, 1997; Solomon et al., 2000; Dean et al., 2001, 2002; Solomon and Dean, 2002).

Gap junctions are formed by proteins that create intercellular channels; these transmembrane pores facilitate the direct exchange of small molecules, metabolites, adenosine triphosphate (ATP) and ions between cells in direct contact (Hooper and Subak-Sharpe, 1981; Loewenstein, 1981; Picoli et al., 2012). These channels are composed by a combination of two hemichannels (connexons), each connexon containing a group of six proteins called connexins (Cx). These proteins fold in a hexameric structure creating a hydrophilic channel that connects the cytosol of neighboring cells (Davidson and Baumgarten, 1988; Solomon and Dean, 2002; Picoli et al., 2012).

Cells that communicate via electrical synapses, in most cases, express more than one Cx isoform (Solomon and Dean, 2002), thus each connexon may consist of a single or multiple isoforms of Cxs (homomeric or heteromeric connexons, respectively; Bruzzone et al., 1996; Kumar and Gilula, 1996; Laird, 2006). The physiological properties of the gap junction depend on the protein isoforms that are present in the connexon (Kumar and Gilula, 1996; Bruzzone and Ressot, 1997), which can explain the differences in sensitivity to intracellular pH (pHi), intracellular calcium and phosphorylation (Goodenough et al., 1996). Among the 15 connexin isoforms identified in the CNS of adult and newborn rats (Beyer et al., 1990; Dermietzel and Spray, 1993; Bruzzone et al., 1996; Dahl, 1996; Condorelli et al., 1998), Cxs 26, 32, and 36 are the most abundantly expressed in neuronal cells (Dermietzel et al., 1989; Belliveau et al., 1991; Belliveau and Naus, 1995; Condorelli et al., 1998; Solomon et al., 2001).

A striking characteristic of the LC neurons is the cell-to-cell coupling via gap junctions (Christie et al., 1989; Christie and Jelinek, 1993; Travagli et al., 1995; Ishimatsu and Williams, 1996; Oyamada et al., 1999; Alvarez-Maubecin et al., 2000; Ballantyne and Scheid, 2000). The cellular coupling facilitates the communication between neurons and it is important to coordinate the firing of the LC neurons, since it seems that the entire nucleus fires simultaneously in response to sensory inputs (Nestler et al., 1999). In fact, this synchronism is important for increase of NA release from LC neurons (Christie et al., 1989; Travagli et al., 1995; Ishimatsu and Williams, 1996; Alvarez-Maubecin et al., 2000). According to Rash et al. (2007), the LC neurons of adult rats express Cx32 and Cx36, while the presence of Cx26, an isoform which is sensitive to CO_2_, is still controversial topic in the field (Alvarez-Maubecin et al., 2000). Previous studies have shown that Cx36 expression in the CNS increases during the first week of life and reduces in adults (Sohl et al., 1998; Belluardo et al., 2000; Solomon, 2003). This Cx participates in the astrocytic and neuronal differentiation during development (Hartfield et al., 2011) and also plays an important role in learning and memory consolidation (Frisch et al., 2005). Regarding Cx32, this isoform is only detected between 5 and 10 days postnatal (Dermietzel et al., 1989; Belliveau et al., 1991; Belliveau and Naus, 1995; Nadarajah et al., 1997; Solomon et al., 2001) and is hypothesized to participate in central CO_2_ chemoreception (Solomon et al., 2001). Further studies are needed to clarify the role of Cx36 and Cx32 in the LC.

Oyamada et al. (1998) demonstrated that transversal brainstem slices of newborn rats presented 83% of LC neurons being modulated by the respiratory rhythm and this modulation depends on the activation of an excitatory amino acid pathway. Also there are evidences that LC neurons exhibit spontaneous depolarizations that do not depend on the chemical synaptic transmission (Williams and Marshall, 1987; Dean et al., 2001) and this electrical synchronization is interrupted when a gap junction blocker is administered (Christie et al., 1989; Travagli et al., 1995; Ishimatsu and Williams, 1996; Oyamada et al., 1999; Alvarez-Maubecin et al., 2000; Ballantyne and Scheid, 2000; Andrzejewski et al., 2001; Ballantyne et al., 2004), reinforcing the role of the electrical coupling in the maintenance of endogenous rhythmic activity of LC neurons (Hayashida et al., 2010).

Recently, Nichols et al. (2008) demonstrated using newborn brainstem slices that carbenoxolone (CARB, a gap junction blocker) decreases the percentage of LC neurons that are responsive to CO_2_, as well as the magnitude of this response (chemosensitivity index – CI) during postnatal development. In addition, Rash et al. (2007) reported that LC neurons are strongly electrically coupled during early post-natal development, suggesting that gap junctions may play an important role in the chemosensitive response of LC neurons during development. However, the function of gap junctions may differ among other chemosensitive nuclei, since the synaptic blockade of NTS neurons by CARB perfusion reduces the ventilatory response to hypercapnia in 7–10 week-old awake rats but not in >10-week-old rats (Parisian et al., 2004). In the RTN, CARB perfusion decreases the ventilatory response to CO_2_ in younger animals, but increases the hypercapnic ventilatory response in older animals (Hewitt et al., 2004).

The pattern of development regarding the ventilatory response to CO_2_, present a clear and important change in the ventilatory response to hypercapnia during the neonatal period in rats, especially when comparing young *versus* older animals (Putnam, 2001). The LC of rats younger than 10 days (P10) has a high percentage of chemosensitive neurons (70–80%) and the magnitude of the response to hypercapnia is high (approximately 235%) compared with older neonates (125%; Hartzler et al., 2007). Additionally, the percentage of LC neurons and the CI is not affected by CARB in rats younger than P10 (Hartzler et al., 2007; Nichols et al., 2008). On the other hand, the CO_2_ chemosensitive response of LC neurons of older neonates (>P10) is reduced by 20% in the presence of CARB, without affecting the CI, suggesting that the electrical coupling increases the responsiveness to CO_2_ of LC neurons in newborn rats (Hartzler et al., 2007; Nichols et al., 2008; Gargaglioni et al., 2010).

**FIGURE 1:**
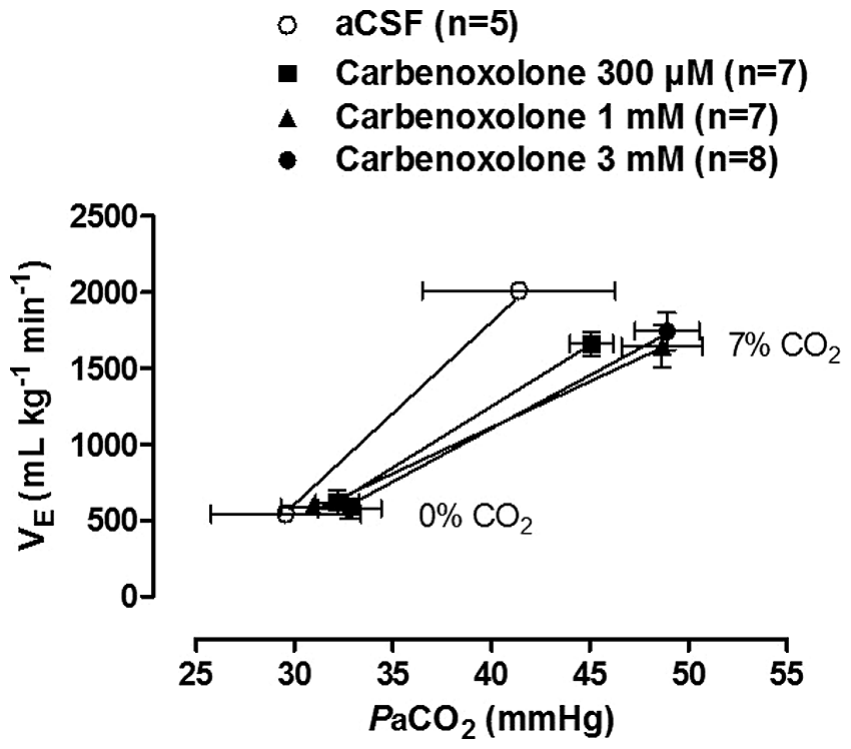
**Effect of bilateral intra-LC microinjections of vehicle (aCSF/100 nL) or carbenoxolone (300 μM, 1 mM, or 3 mM/100 nL) on CO_2_ sensitivity (relationship between and PaCO_2_).** Values are expressed as mean ± SEM. The 1 and 3 mM carbenoxolone sensitivity curves presented lower slopes than the vehicle curve (*P* < 0.01). With permission from Elsevier (Patrone et al., 2014).

A recent study by our group investigated the participation of gap junctions in the CO_2_ ventilatory response in unanesthetized adult rats by bilaterally microinjecting CARB into the LC of Wistar rats (Patrone et al., 2014). During normocapnic conditions, the gap junctions have no regulatory role on ventilation, since CARB microinjection did not change the resting ventilation (Patrone et al., 2014). Regarding hypercapnic ventilatory response, our findings corroborate the literature since CARB (1 mM or 3 mM) microinjection into LC neurons resulted in a significant reduction, approximately 24 and 20%, respectively, in the ventilatory response to 7% CO_2_. This result was confirmed by the lower slopes of the 1 and 3 mM CARB CO_2_ sensitivity curves compared to the curve for vehicle-injected rats (**Figure 1**). Therefore, our data suggest that gap junctions in the LC are important for modulation of the CO_2_ drive to breathe in adult rats.

Recent studies have also addressed whether LC electrical synapses are involved in cardiovascular regulation. Microinjection of CARB in LC did not affect cardiovascular parameters during normocapnia, suggesting that gap junctions in LC neurons are unlikely to play a role in the tonic control of cardiovascular function. However, heart rate decreased after CO_2_ exposure in the group treated with 3 mM CARB, indicating a possible role of LC neuronal gap junctions in the regulation of heart rate during CO_2_ challenge. Summarizing, electrical synapses in LC neurons, specifically through gap junctions, play a role in the CO_2_ drive to breathe and also modulate heart rate under hypercapnic conditions.

## NEUROCHEMICAL MODULATION OF THE LC

### GLUTAMATE

Glutamate is an endogenous amino acid that acts as a major excitatory neurotransmitter in the mammalian CNS (Ruggiero et al., 2011) and participates in the central generation and transmission of respiratory rhythm (Bonham, 1995). Glutamate receptors are divided into two subtypes, ionotropic and metabotropic. Ionotropic receptors can be further divided in NMDA (N-methyl-D-aspartate) and non-NMDA (AMPA and Kainate). The NMDA receptors bind simultaneously with glutamate and glycine, resulting in the influx of Na^+^ and Ca^2+^, whereas the non-NMDA receptors are more rapidly depolarized, causing a Na^+^ influx (Bowie, 2008). The metabotropic receptors are G-protein coupled, and they can be divided in mGlu I, II, and III. These receptors appear to be related with presynaptic regulation, and they also modulate the transmission of the respiratory rhythm to phrenic motoneurons; however, they are not involved in respiratory rhythmogenesis (Liu et al., 1990).

Glutamate is a primary excitatory neurotransmitter in the LC (Singewald and Philippu, 1998), and several studies identified different subunits of ionotropic glutamate receptors, with the majority belonging to the non-NMDA category (Sato et al., 1993; Wisden and Seeburg, 1993; Petralia et al., 1994). Experiments using anesthetized rabbits demonstrated that activation of LC neurons with L-glutamate increased the respiratory frequency and discharge rate of the phrenic nerve, while decreasing duration of inspiration and expiration (Li et al., 1992).

The major glutamatergic afferents to the LC come from the lateral nucleus paragigantocellularis (Ennis et al., 1992). Another important source of glutamate inputs are neurons localized in the lateral hypothalamus, which are also responsible for producing orexin (Peyron et al., 1998). A previous study performed by Henny et al. (2010) demonstrated that a subset of orexinergic terminals have the ability to release glutamate in addition to orexin, and do play a role in postsynaptic targets via glutamatergic synapses, including terminals in the LC. More recently, it was demonstrated that catecholaminergic neurons of the rostral ventrolateral medulla (RVLM), most likely C1 neurons, establish a glutamatergic synapse with LC (Holloway et al., 2013). LC noradrenergic neurons are important for wake-promoting systems; therefore, glutamatergic signaling and LC neurons could be important to the state-dependent control of breathing (Gonzalez and Aston-Jones, 2006).

**FIGURE 2:**
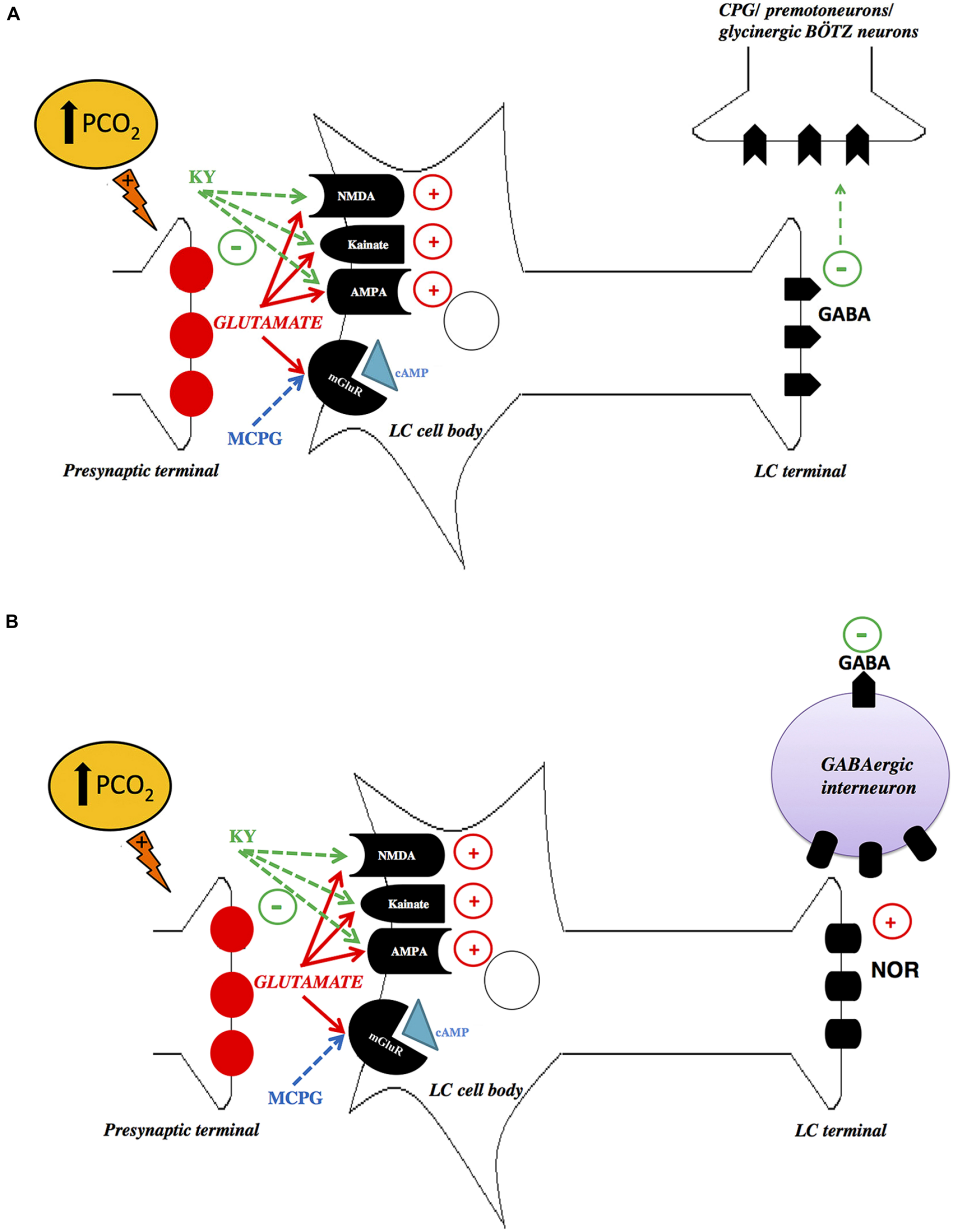
**Schematic drawings depicting possible mechanisms of glutamatergic signaling in the locus coeruleus (LC). (A)** Hypercapnia increases glutamate release in the LC, which directly activates LC gabaergic neurons. Inhibition of NMDA, AMPA, and Kainate receptors by KY may inhibit LC GABAergic neurons, which project to the central respiratory pattern generator (CPG), or directly to the respiratory premotoneurons, causing an increase in the activity of these neurons, promoting an increase in ventilation. **(B)** Another possibility is that hypercapnia increases glutamate release in the LC, which possibly activates noradrenergic neurons that induce GABA release. Inhibition of NMDA, AMPA and Kainate receptors by KY may decrease the release of excitatory neurotransmitter of LC, probably noradrenaline (NOR), leading to inhibition of GABAergic interneuron, which in turn may reduce the activation of the glycinergic Bötzinger (BÖTZ) neurons, causing an increase in the hypercapnic ventilatory response. LC, locus coeruleus; PGC, nucleus paragigantocellularis; KY, kynurenic acid; MCPG, α-methyl-4-carboxyphenylglycine. With permission from Wiley Online Library (Taxini et al., 2013).

Recently, we have investigated the role of glutamatergic inputs in the LC modulation of the ventilatory response to hypercapnia in unanesthetized rats by applying Kynurenic Acid (KY, an antagonist at ionotropic glutamate receptors) or α-methyl-4-carboxyphenylglycine (MCPG, an antagonist at metabotropic glutamate receptors; Taxini et al., 2013). Microinjections of MCPG did not affect cardiorespiratory responses during normocapnia or hypercapnia. Remarkably, microinjection of KY into the LC amplified pulmonary ventilation during 7% CO_2_, indicating that glutamatergic inputs on LC neurons cause an inhibition of the hypercapnia-induced hyperpnea. Our results are comparable to previous evidence in the literature that glutamate acts on ionotropic glutamatergic receptors in the LC and RVLM, thus inhibiting the ventilatory response to hypoxia (Dillon et al., 1991; Ferreira et al., 2004). Despite the fact that glutamate is classically known as an excitatory neurotransmitter in the CNS, there is also evidence that glutamate acts on NMDA receptors exerting a “functionally inhibitory” role, by suppressing circuit-level neural activity through the activation of GABAergic interneurons (Fitzgerald, 2012). Thus, inhibition of NMDA receptors by antagonist drugs may activate GABAergic neurotransmission as observed in rat prefrontal cortex (Homayoun and Moghaddam, 2007). Presumably, the increased ventilatory response to CO_2_ after NMDA blockade could be linked to inhibition of inhibitory interneurons presents in the LC, which accounts for approximately 8% of LC neurons (Iijima and Ohtomo, 1988; de Carvalho et al., 2010). A second possible explanation is that the projections from the LC to the central respiratory pattern generator, or directly to the respiratory premotoneurons, may be mediated by LC GABAergic neurons (**Figure 2**). Therefore, the attenuation of the hypercapnic ventilator response, mediated by LC ionotropic receptors, can be the result of excitatory projections to glycinergic Bötzinger (BÖTZ) neurons, which participates in the termination of inspiration (Champagnat et al., 1982; Bonham, 1995; Schreihofer et al., 1999). Thus, a possible role of LC glutamatergic mechanisms could be in limiting the extent of hypercapnia-induced hyperpnea. However, the interaction between the LC neurons and the glycinergic BÖTZ neurons is still unclear.

With respect to cardiovascular regulation, glutamatergic receptor antagonism did not affect blood pressure and heart rate during normocapnia and hypercapnia, supporting previous findings which demonstrate that the inhibition of LC neurons (Sved and Felsten, 1987; Miyawaki et al., 1991) or electrolytic lesions of this nucleus (Anselmo-Franci et al., 1998) have no effect on cardiovascular parameters.

### SEROTONIN

Serotonin (5-hydroxytryptamine, 5-HT) is a neurotransmitter involved in many physiological functions such as the sleep–wake cycle, mood, appetite, and neurovegetative control. Serotonin can also be linked to emotional disorders such as anxiety and depression (Jacobs and Azmitia, 1992; Brown and Gershon, 1993; Jouvet, 1999; Saper et al., 2001; Jones, 2005). Serotoninergic neurons are located mainly in the raphe nuclei and project to almost all brain areas and the spinal cord (Törk, 1990; Baumgarten and Göthert, 1997).

Extensive reciprocal projections are reported between raphe and LC neurons (Pasquier et al., 1977; Pickel et al., 1977; Cedarbaum and Aghajanian, 1978; Morgane and Jacobs, 1979; Baraban and Aghajanian, 1981; Vertes and Kocsis, 1994; Luppi et al., 1995; Peyron et al., 1996; Kim et al., 2004). In fact, LC neurons receive dense serotonergic projections coming mostly from dorsal raphe (DR) and pericoerulear 5-HT neurons (Aston-Jones et al., 1991a; Kaehler et al., 1999; Kim et al., 2004), and these projections are important for controlling sleep–wake cycle, vigilance, analgesia, cognition, depression, pain and anxiety (Redmond and Huang, 1979; Segal, 1979; Charney and Redmond, 1983; Uhde et al., 1984; Mokha et al., 1985; Meltzer and Lowy, 1987; Seiver, 1987; Kim et al., 2004). Stimulation of central 5-HT neurons causes reductions in the LC spontaneous and pain-evoked activity (Segal, 1979) and 5-HT, when applied in the LC, attenuates the excitatory responses of this nucleus to sensorial, neurochemical and electrical stimuli (Bobker and Williams, 1989; Aston-Jones et al., 1991a).

Administration of the 5-HT_1A_ receptor antagonist WAY-100635 in anesthetized rats suppressed LC activity. Interestingly, this effect can be prevented by lesioning central 5-HT neurons (injection of 5,7 DHT intracerebroventricularly) or by 5-HT_2_ receptor antagonism, indicating that the suppressant effect of WAY-100635 on noradrenergic neuron firing is dependent on intact 5-HT neurons and also involves an over-activation of excitatory 5-HT_2_ receptors in inhibitory neurons projecting to LC (Haddjeri et al., 1997; Szabo and Blier, 2001).

Recently, we have demonstrated that hypercapnia induces an increase in 5-hydroxyindole-3-acetic acid (5-HIAA) levels and serotonergic turnover (5-HTTIA/5-HT ratio) in the LC of rats (de Souza Moreno et al., 2010). In this study, we also microinjected WAY-100635, Ketanserin (5-HT_2A_ antagonist), or DOI (5-HT_2A_ agonist) into the LC of unanesthetized rats to verify the role of 5-HT receptors in the CO_2_ ventilatory response. Antagonism of 5-HT receptors in the LC had no effect on resting breathing parameters (de Souza Moreno et al., 2010). Microinjection of WAY-100635 into the LC decreased the ventilatory response to CO_2_, possibly due to inhibition of noradrenergic neurons. The reduction of the response to CO_2_ by WAY-100635 may be due to its direct antagonism of presynaptic 5-HT_1A_ receptors, leading to an increase in 5-HT release and consequential activation of postsynaptic 5-HT_2A_ receptors in the LC.

Another possible explanation for our results is that WAY-100635 may decrease the glutamate evoked activation of LC neurons. In this regard, Aston-Jones et al. (1991a) has previously demonstrated that 5-HT decreased glutamate evoked activation of LC cells using extracellular recordings from single neurons in an anesthetized preparation. Moreover, Bobker and Williams (1989) showed that the receptors mediating this inhibition are the 5-HT_1A_ and 5-HT_1B_ subtypes, probably located presynaptically in the PGi terminals, since LC receives afferent excitatory glutamatergic inputs from the PGi nucleus (Ennis and Aston-Jones, 1987). Therefore, this interaction between glutamate and 5-HT may play a role in preventing a strong inhibition of LC during hypercapnia.

In fact, microinjections of Ketanserin in the LC increased the hypercapnic hyperpnea, whereas the 5-HT_2A_ receptor agonist DOI evoked an opposite effect. Therefore, we suggested that release of 5-HT is increased in the LC during CO_2_ challenge and that 5-HT negatively modulates the LC stimulatory role in the hypercapnic ventilatory response, probably acting through postsynaptic 5-HT_2A_ receptors or presynaptic 5-HT_2A_ in GABAergic terminals. The possible action of 5-HT on GABAergic terminals corroborates previous study that reported that GABA_A_ receptor antagonist bicuculline on LC neurons blocked the inhibitory effects of intravenously injected DOI on LC firing (Chiang and Aston-Jones, 1993).

### ATP

ATP is an important intracellular energy source for all cells and is present in all mammalian neurons (Edwards and Gibb, 1993). Burnstock (1972) proposed the concept of purinergic signaling, after the observation that a purine nucleotide could act as a neurotransmitter in non-adrenergic, non-cholinergic (NANC) nerves supplying the gut and urinary bladder. Currently, it is established that ATP acts as a sole neuromodulator or is co-released with other neuromodulators in the peripheral and in the CNS (Abbracchio et al., 2009).

In the CNS, ATP acts on regions involved in cardiovascular and respiratory regulation, including the LC (Thomas and Spyer, 2000; Thomas et al., 2001; Gourine et al., 2002, 2005; Antunes et al., 2005; Yao and Lawrence, 2005). LC neurons are of particular interest as purinergic signaling targets because ATP can act as a neuromodulator on neurons terminating within the LC or as a co-transmitter of recurrent axonal collaterals or dendrites of intrinsic LC neurons (Tschöpl et al., 1992; Nieber et al., 1997; Poelchen et al., 2001). Poelchen et al. (2001) reported for the first time that ATP and NA are co-released from recurrent axon collaterals onto LC neurons producing biphasic synaptic potentials consisting of early depolarizing (epsp = excitatory postsynaptic potential) and late hyperpolarizing (ipsp = inhibitory postsynaptic potential) components, respectively.

P2 purinergic receptors are activated by ATP (Burnstock, 1997) and are classified into P2X (ligand-activated cationic channel, ionotropic receptor) and P2Y (G-protein-coupled receptor, metabotropic receptor) subtypes (Abbracchio and Burnstock, 1994; Fredholm et al., 1994; Mateo et al., 1998). The LC contains more P2X receptors, and the P2X population consists mainly of the P2X2, P2X3, and P2X6 subtypes (Yao et al., 2000). Although P2X7 receptors have been found in LC area and it appeared that these receptors are located in astrocytes (Khakpay et al., 2010). A short stimulation of the P2X7 receptors leads to activation of cationic currents as the other subtypes of P2X receptors although a repeated or prolonged ATP exposure also opens a large pore which allows the passage of organic cations and dye molecules (Virginio et al., 1999).

ATP evokes the release of NA (Poelchen et al., 2001) and ATP within LC, when acting on presynaptic P2X receptors (Boehm, 1999), and inhibits the release of NA and ATP, when acting on presynaptic P2Y receptors (von Kügelgen et al., 1994, 1995; Poelchen et al., 2001). In LC neurons, activity of NA on postsynaptic α2-adrenoceptors has been shown to mediate hyperpolarization (Aghajanian and Vandermaelen, 1982; Poelchen et al., 2001), while activation of both P2X and P2Y receptors by ATP has been shown to depolarize LC neurons (Harms et al., 1992; Shen and North, 1993; Scheibler et al., 2004). Thus, ATP may be released to provide an excitatory counterbalance to the inhibitory noradrenergic drive of these neurons (Illes et al., 1994).

There is evidence that tyrosine hydroxylase (TH) co-localizes with P2X receptors in the LC (Yao et al., 2000) indicating the presence of P2X receptors in LC noradrenergic neurons. Moreover, *α,β*-meATP (a P2X receptor agonist) excites LC neurons in *in vitro* preparations of the pons (Tschöpl et al., 1992; Shen and North, 1993), increasing the frequency of spontaneous action potentials in LC (Tschöpl et al., 1992).

On the other hand, Kuwahata (2004) demonstrated that ATP produced a hyperpolarizing response or a transient hyperpolarizing response that preceded the depolarization in LC neurons. However, AMP-PNP (adenosine 5^′^-(β,γ-imido) triphosphate, which is more resistant to dephosphorylation than ATP, only depolarizes LC neurons. According to Kuwahata (2004) adenosine is the likely source of the ATP-induced hyperpolarizing response in LC neurons.

Despite the excitatory effects of ATP in LC neurons, little is known about the physiological consequences of ATP when administered in the LC *in vivo,* with antinociception being an exception (Fukui et al., 2004). A recent study by our group focused on the role of ATP in the cardiorespiratory control through P2 receptor-mediated mechanisms in the LC of unanesthetized rats (Biancardi et al., 2014). In this study we provided the first functional evidence that purinergic neuromodulation within the LC is important for cardiorespiratory control in unanesthetized animals. In normocapnic conditions, ATP release within the LC may occur to maintain respiratory frequency (Biancardi et al., 2014). We observed that PPADS (ATP antagonist with predominant actions on P2X receptors) decreased respiratory frequency, whereas suramin (P2 non-selective antagonist) did not change any of the investigated respiratory variables (Biancardi et al., 2014), when injected into the LC.

Microinjections of ATP and the P2X receptor agonist *α,β*-meATP into brainstem respiratory nuclei, such as the *nucleus tractus solitarius* (NTS) and RVLM, also cause changes in respiratory amplitude and frequency (Thomas et al., 2001; Antunes et al., 2005). Several studies have shown that hypercapnia triggers ATP release in the RVLM (for review see Gourine et al., 2005). The injection of P2X receptor antagonists in this region reduces the ventilatory response to hypercapnia, indicating that ATP, acting on P2X receptors, plays a critical role in the chemoreception to CO_2_/pH (Thomas and Spyer, 2000; Gourine et al., 2005).

Biancardi et al. (2008) also observed that the ablation of about 80% of LC noradrenergic neurons was associated with a large decrease (64%) in the ventilatory response to increased CO_2_. More recently, we observed that microinjections of *α,β*-meATP into the LC promoted a greater increase in ventilation during the hypercapnic challenge (7% CO_2_), compared with the controls (Biancardi et al., 2014). This was blocked by pretreatment with PPADS, indicating that *α,β*-meATP is acting on P2X receptors to evoke that effect. Thus, LC purinergic signaling appears to play a role in the CO_2_ drive to breathe, specifically through the activation of P2 receptors, accelerating the ventilatory response to hypercapnia. This observation was probably due to P2X activation of noradrenergic neurons, triggering further NA and ATP release which in turn activate rhythm-generating circuits and medullary respiratory neurons in one or more of the following brainstem nuclei: RTN, RVLM, prepositus hypoglossi nucleus, and NTS (Aston-Jones et al., 1986, 1991b; Astier et al., 1990; Pieribone and Aston-Jones, 1991; Vulchanova et al., 1997; Llewellyn-Smith and Burnstock, 1998; Kanjhan et al., 1999; Thomas and Spyer, 2000; Yao et al., 2000; Gourine et al., 2003; Lorier et al., 2007, 2008; Huxtable et al., 2009).

Unexpectedly, suramin induced an increase in ventilation during CO_2_ exposure. However, this effect occurred 20 min after the microinjections, while the effect of the P2X agonist appeared only 2 min after microinjections. These observations suggest that suramin may be acting on presynaptic P2Y receptors, which, due to location and metabotropic mechanism of activation, may introduce a delay in the response. Given that these receptors are G-protein coupled (Abbracchio and Burnstock, 1994), the latency to induce neurotransmitter release is prolonged.

Yao and Lawrence (2005) suggested that ATP modulates the cardiovascular system via activation of P2X receptors in the LC of anesthetized rats. Moreover, they hypothesize that purinergic and noradrenergic systems are tonically active within the LC and interact functionally. They also demonstrated that microinjections of a P2X receptor agonist into the LC induced hypotension and bradycardia in anesthetized animals. Conversely, microinjections of P2 receptor antagonists induced hypertension and tachycardia. In another study, Biancardi et al. (2014) evaluated the role of ATP in the modulation of cardiovascular responses to CO_2_ exposure. They observed that both PPADS and suramin induced an increase in mean arterial pressure (MAP) under hypercapnic conditions, although *α,β*-MeATP did not change cardiovascular parameters in unanesthetized animals. This increase in MAP may be due to a blockade of P2X and P2Y postsynaptic receptors, which may attenuate depolarization of LC noradrenergic neurons, thereby reducing NA release. A decrease in NA, in turn, decreases RVLM inhibition and promotes pressor response.

**FIGURE 3:**
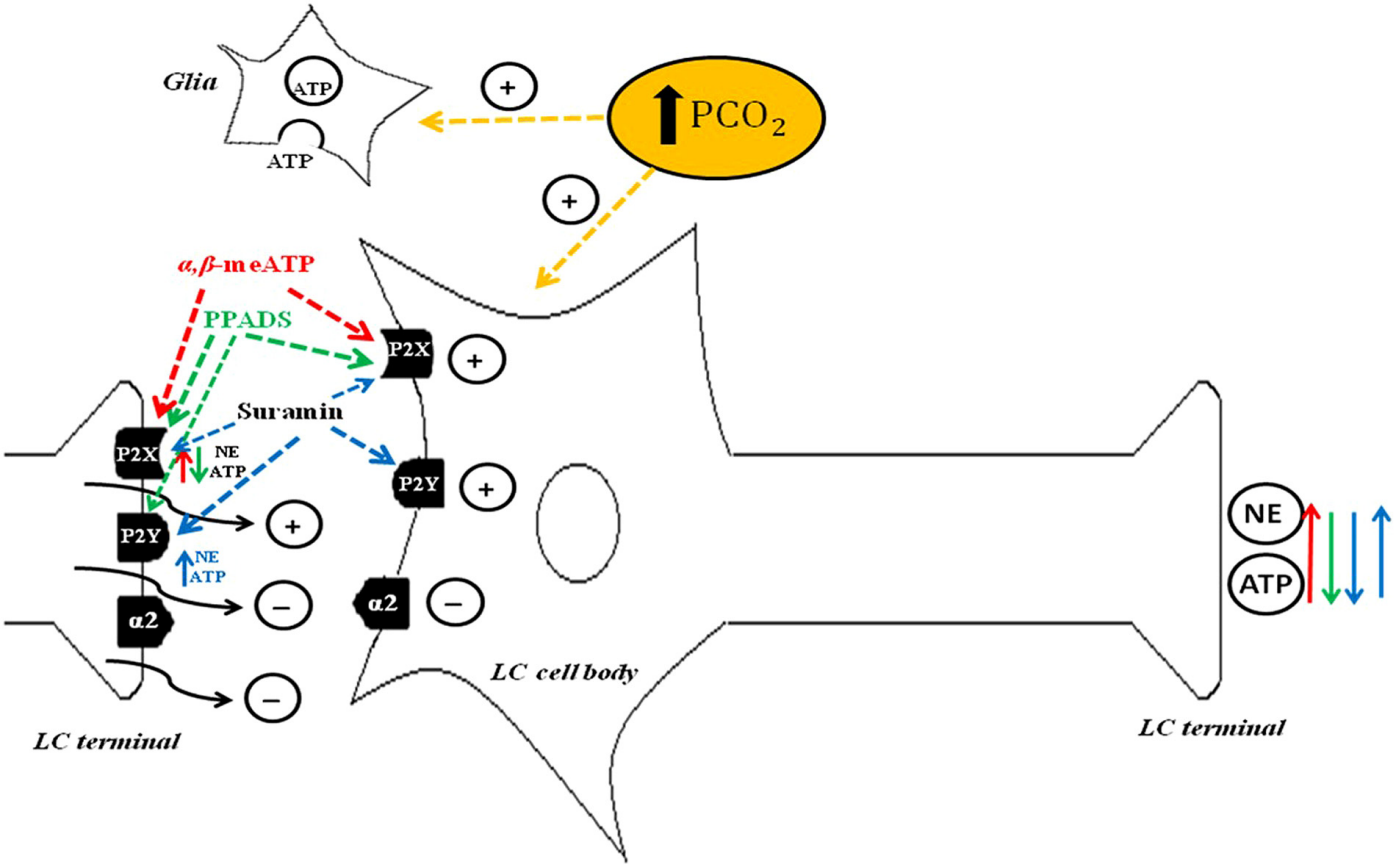
**Schematic drawing depicting possible mechanisms of purinergic signaling in the locus coeruleus (LC).** ATP induces depolarization of noradrenergic neurons through P2X and P2Y postsynaptic receptors (Harms et al., 1992; Shen and North, 1993), NE and ATP release through P2X presynaptic receptors (Boehm, 1999) and inhibition of NE and ATP release through P2Y presynaptic receptors (von Kügelgen et al., 1994, 1995; Poelchen et al., 2001). α,β-meATP agonism of P2X presynaptic receptors may promote NE and ATP release, whereas agonism of P2X postsynaptic receptors may activate noradrenergic neurons to further increase NE and ATP release (red arrow, Nieber et al., 1997). PPADS blocks mainly P2X receptors, which reduces NE and ATP release at LC terminals (green arrow). Suramin blocks P2X and P2Y receptors. The postsynaptic receptors reduce neurotransmitter release (blue down arrow). The presynaptic P2Y receptor subtype increases NE and ATP release, thereby further activating noradrenergic neurons and causing additional NE and ATP release (blue up arrow). PCO_2_ increase in the cerebrospinal fluid activates LC noradrenergic neurons (yellow arrow; Biancardi et al., 2008) and astrocytes (yellow arrow; Alvarez-Maubecin et al., 2000; Spyer et al., 2004) which, in turn, release ATP. With permission from Wiley Online Library (Biancardi et al., 2014).

**Figure 3** depicts proposed LC purinergic neuromodulation mechanisms based on Biancardi et al. (2014) and evidence from Williams et al. (1985), Aghajanian and Wang (1986), Harms et al. (1992), Shen and North (1993), von Kügelgen et al. (1994), von Kügelgen et al. (1995), Nieber et al. (1997), Boehm (1999), Alvarez-Maubecin et al. (2000), Poelchen et al. (2001), Spyer et al. (2004), and Biancardi et al. (2008).

Until recently, there were no studies available in the literature regarding ATP-mediated respiration mechanisms in the LC. However, a recent study from our group (Biancardi et al., 2014) introduced the idea of the participation of purinergic signaling in the LC in the modulation of respiration. Further investigation is needed, nevertheless, to address how hypercapnic challenges modulate purinergic signaling within the LC. An increase in arterial blood PCO_2_ triggers immediate ATP release from ventral chemosensory site(s) on the surface of the medulla, and glial cells appear to be the likely source of this ATP release in response to such stimuli (Gourine et al., 2005). The striking abundance of astrocytes in the LC (Alvarez-Maubecin et al., 2000) supports the idea that these cells may play a distinctive role in that nucleus. Based on the reviewed literature, we believe that the field would benefit greatly from further investigation of the possibility that hypercapnic exposure induces ATP release from LC astrocytes, which contribute to modulation of respiratory activity.

## CONCLUSION

We have reviewed evidence that LC neurons exert an important role in hypercapnic ventilatory response, and this response is chemically modulated by 5-HT, glutamate, ATP and electrically controlled by GAP junctions. As it is well known, LC is an important pontine group that has access to respiration-related cell groups in the caudal brainstem, and also modulates the sleep wake–cycle and many other homeostatic function. Therefore, this nucleus can be considered a gateway to adjust respiration to sleep–wake events. In addition, LC has been linked to pathological conditions such as Rett syndrome, where individuals present decreased CO_2_ chemosensitivity. This CNS dysfunction is characterized by the loss of function mutations in the X-linked gene encoding in Methyl-CpG-binding protein-2 (MeCP2; Amir et al., 1999). MeCP2 knockout mice presents morphological (decreased soma size), electrical (reduced membrane conductance and strong afterhyperpolarization amplitude), and neurochemical (reduced TH content) alterations of LC neurons (Taneja et al., 2009). However, the neurobiological mechanisms underlying such breathing disorders presented by people with Rett syndrome are still unclear. According to Taneja et al. (2009), LC is a critical site at which loss of MeCP2 results in CNS dysfunction and restoration of normal LC function may attenuate the symptoms of this syndrome. Therefore, a better comprehension of LC and its modulators in breathing control is needed to clarify this issue.

## Conflict of Interest Statement

The authors declare that the research was conducted in the absence of any commercial or financial relationships that could be construed as a potential conflict of interest.

## References

[B1] Abbracchio M. P. Burnstock G. (1994). Purinoceptors: are there families of P2X and P2Y purinoceptors? *Pharmacol. Ther.* 64 445–475 10.1016/0163-7258(94)00048-4 7724657

[B2] Abbracchio M. P. Burnstock G. Verkhratsky A. Zimmermann H. (2009). Purinergic signalling in the nervous system: an overview. *Trends Neurosci.* 32 19–29 10.1016/j.tins.2008.10.001 19008000

[B3] Aghajanian G. K. Vandermaelen C. P. (1982). α2-Adrenoceptor-mediated hyperpolarization of locus coeruleus neurons: intracellular studies in vivo. *Science* 215 1394–1396 10.1126/science.6278591 6278591

[B4] Aghajanian G. K. Wang Y. Y. (1986). Pertussis toxin blocks the outward currents evoked by opiate and α 2-agonists in locus coeruleus neurons. *Brain Res.* 371 390–394 10.1016/0006-8993(86)90382-3 3697768

[B5] Alvarez-Maubecin V. Garcia-Hernandez F. Williams J. T. Van Bockstaele E. J. (2000). Functional coupling between neurons and glia. *J. Neurosci.* 20 4091–4098 10818144 10.1523/JNEUROSCI.20-11-04091.2000 PMC6772654

[B6] Amir R. E. Van den Veyver I. B. Wan M. Tran C. Q. Francke U. Zoghbi H. Y. (1999). Rett syndrome is caused by mutations in X-linked MECP2, encoding methyl-CpG-binding protein 2. *Nat. Genet.* 23 185–188 10.1038/13810 10508514

[B7] Andrzejewski M. Muckenhoff K. Scheid P. Ballantyne D. (2001). Synchronized rhythms in chemosensitive neurones of the locus coeruleus in the absence of chemical synaptic transmission. *Respir. Physiol.* 129 123–140 10.1016/S0034-5687(01)00300-0 11738650

[B8] Anselmo-Franci J. A. Peres-Polon V. L. da Rocha-Barros V. M. Moreira E. R. Franci C. R. Rocha M. J. (1998). C-fos expression and electrolytic lesions studies reveal activation of the posterior region of locus coeruleus during hemorrhage induced hypotension. *Brain Res.* 799 278–284 10.1016/S0006-8993(98)00468-5 9675311

[B9] Antunes V. R. Bonagamba L. G. Machado B. H. (2005). Hemodynamic and respiratory responses to microinjection of ATP into the intermediate and caudal NTS of awake rats. *Brain Res.* 1032 85–93 10.1016/j.brainres.2004.10.048 15680945

[B10] Astier B. Van Bockstaele E. J. Aston-Jones G. Pieribone V. A. (1990). Anatomical evidence for multiple pathways leading from the rostral ventrolateral medulla (nucleus paragigantocellularis) to the locus coeruleus in rat. *Neurosci. Lett.* 118 141–146 10.1016/0304-3940(90)90612-D 2274260

[B11] Aston-Jones G. Akaoka H. Charléty P. Chouvet G. (1991a). Serotonin selectively attenuates glutamate-evoked activation of noradrenergic locus coeruleus neurons. *J. Neurosci.* 11 760–769 1672153 10.1523/JNEUROSCI.11-03-00760.1991 PMC6575362

[B12] Aston-Jones G. Shipley M. T. Chouvet G. Ennis M. Van Bockstaele E. Pieribone V. (1991b). Afferent regulation of locus coeruleus neurons: anatomy, physiology and pharmacology. *Prog. Brain Res.* 88 47–75 10.1016/S0079-6123(08)63799-1 1687622

[B13] Aston-Jones G. Ennis M. Pieribone V. A. Nickell W. T. Shipley M. T. (1986). The brain nucleus locus coeruleus: restricted afferent control of a broad efferent network. *Science* 234 734–737 10.1126/science.3775363 3775363

[B14] Aston-Jones G. Foote S. L. Bloom F. E. (1984). “Anatomy and physiology of locus coeruleus neurons: functional implications,” in *Norepinephrine* (*Frontiers of Clinical Neuroscience*) Vol. 2 eds Ziegler M. Lake C. R. (Baltimore:Williams and Wilkins) 92–116

[B15] Aston-Jones G. Shipley M. T. Grzanna R. (1995). “The Locus coeruleus, A5 and A7 noradrenergic cell groups,” in *The Rat Nervous System* ed. Paxinos G. (Boca Raton:Academic Press) 183–213

[B16] Ballantyne D. Andrzejewski M. Mückenhoff K. Scheid P. (2004). Rhythms, synchrony and electrical coupling in the Locus coeruleus. *Respir. Physiol. Neurobiol.* 143 199–214 10.1016/j.resp.2004.07.018 15519556

[B17] Ballantyne D. Scheid P. (2000). Mammalian brainstem chemosensitive neurones: linking them to respiration *in vitro.* *J. Physiol. (Lond.)* 525 567–577 10.1111/j.1469-7793.2000.00567.x 10856112 PMC2269968

[B18] Baraban J. M. Aghajanian G. K. (1981). Noradrenergic innervation of serotonergic neurons in the dorsal raphe: demonstration by eléctron microscopic autoradiography. *Brain Res.* 204 1–11 10.1016/0006-8993(81)90646-6 6166350

[B19] Baumgarten H. G. Göthert M. (1997). “Serotoninergic neurons and 5-HT receptors in the CNS,” in *Handbook of Experimental Pharmacology* Vol. 129. Berlin:Springer

[B20] Belliveau D. J. Kidder G. M. Naus C. C. G. (1991). Expression of gap junction genes during postnatal neural development. *Dev. Genet.* 12 308–317 10.1002/dvg.1020120408 1657468

[B21] Belliveau D. J. Naus C. C. G. (1995). Cellular localization of gap junction mRNAs in developing rat brain. *Dev. Neurosci.* 17 81–96 10.1159/000111277 7555741

[B22] Belluardo N. Mudo G. Trovato-Salinaro A. Le Gurun S. Charollais A. Serre-Beinier V. (2000). Expression of connexin36 in the adult and developing rat brain. *Brain Res.* 865 121–138 10.1016/S0006-8993(00)02300-3 10814742

[B23] Berridge C. W. Waterhouse B. D. (2003). The Locus coeruleus-noradrenergic system: modulation of behavioral state and state-dependent cognitive processes. *Brain Res. Brain Res. Rev.* 42 33–84 10.1016/S0165-0173(03)00143-7 12668290

[B24] Beyer E. C. Paul D. L. Goodenough D. A. (1990). Connexin family of gap junction proteins. *J. Membr. Biol.* 116 187–194 10.1007/BF01868459 2167375

[B25] Biancardi V. Bícego K. C. Almeida M. C. Gargaglioni L. H. (2008). Locus coeruleus noradrenergic neurons and CO_2_ chemosensitivity. *Pflugers Arch.* 455 1119–1128 10.1007/s00424-007-0338-8 17851683

[B26] Biancardi V. Bícego K. C. Gargaglioni L. H. (2014). ATP in the locus coeruleus as a modulator of cardiorespiratory control in unanaesthetized male rats. *Exp. Physiol.* 99 232–247 10.1113/expphysiol.2013.074195 24058188

[B27] Bobker D. H. Williams J. T. (1989). Serotonin agonists inhibit synaptic potentials in the rat locus ceruleus *in vitro* via 5-hydroxytryptamine1A and 5-hydroxytryptamine1B receptors. *J. Pharmacol. Exp. Ther.* 250 37–43 2526217

[B28] Boehm S. (1999). ATP stimulates sympathetic transmitter release via presynaptic P2X purinoceptors. *J. Neurosci.* 19 737–746 9880594 10.1523/JNEUROSCI.19-02-00737.1999 PMC6782222

[B29] Bonham A. C. (1995). Neurotransmitters in the CNS control of breathing. *Respir. Physiol.* 101 219–230 10.1016/0034-5687(95)00045-F 8606995

[B30] Bowie D. (2008). Ionotropic glutamate receptors and CNS disorders. *CNS Neurol. Disord. Drug Targets* 7 129–143 10.2174/187152708784083821 18537642 PMC2662616

[B31] Breen L. A. Burde R. M. Loewy A. D. (1983). Brainstem connections to the Edinger-Westphal nucleus of the cat: a retrograde tracer study. *Brain Res.* 261 303–306 10.1016/0006-8993(83)90633-9 6831211

[B32] Brown A. S. Gershon S. (1993). Dopamine and depression. *J. Neural. Transm.* 91 75–109 10.1007/BF01245227 8099801

[B33] Bruzzone R. Ressot C. (1997). Connexins, gap junctions and cell–cell signaling in the nervous system. *Eur. J. Neurosci.* 9 1–6 10.1111/j.1460-9568.1997.tb01346.x 9042562

[B34] Bruzzone R. White T. W. Paul D. L. (1996). Connections with connexins: the molecular basis of direct intercellular signaling. *Eur. J. Biochem.* 238 1–27 10.1111/j.1432-1033.1996.0001q.x 8665925

[B35] Burnstock G. (1972). Purinergic nerves. *Pharmacol. Rev.* 24 509–581 4404211

[B36] Burnstock G. (1997). The past, present and future of purine nucleotides as signalling molecules. *Neuropharmacology* 36 1127–1139 10.1016/S0028-3908(97)00125-1 9364468

[B37] Cedarbaum J. M. Aghajanian G. K. (1978). Afferent projections to the rat locus coeruleus as determined by a retrograde tracing technique. *J. Comp. Neurol.* 178 1–16 10.1002/cne.901780102 632368

[B38] Champagnat J. Denavit-Saubie M. Moyanova S. Rondouin G. (1982). Involvement of amino acids in periodic inhibitions of bulbar respiratory neurones. *Brain Res.* 237 351–365 10.1016/0006-8993(82)90447-4 6123370

[B39] Charney D. S. Redmond D. E. (1983). Neurobiological mechanisms in human anxiety. Evidence supporting central noradrenergic hyperactivity. *Neuropharmacology* 22 1531–1536 10.1016/0028-3908(83)90122-3 6142428

[B40] Chiang C. Aston-Jones G. (1993). A 5-hydroxytryptamine2 agonist augments γ-aminobutyric acid and excitatory amino acid inputs to noradrenergic locus coeruleus neurons. *Neuroscience* 54 417–420 10.1016/0306-4522(93)90262-E 8101639

[B41] Christie M. J. Jelinek H. F. (1993). Dye coupling among neurons of the rat Locus coeruleus during postnatal development. *Neuroscience* 56 129–137 10.1016/0306-4522(93)90568-Z 7694183

[B42] Christie M. J. Williams J. T. North R. A. (1989). Electrical coupling synchronizes subthreshold activity in locus coeruleus neurons in vitro from neonatal rats. *J. Neurosci.* 9 3584–3589 2795142 10.1523/JNEUROSCI.09-10-03584.1989 PMC6569893

[B43] Coates E. L. Li A. Nattie E. E. (1993). Widespread sites of brainstem ventilatory chemoreceptors. *J. Appl. Physiol.* 75 5–14 8376301 10.1152/jappl.1993.75.1.5

[B44] Condorelli D. F. Parenti R. Spinella F. Trovato Salinaro A. Belluardo N. Cardile V. (1998). Cloning of a new gap junction gene (Cx36) highly expressed in mammalian brain neurons. *Eur. J. Neurosci.* 10 1202–1208 10.1046/j.1460-9568.1998.00163.x 9753189

[B45] Dahl G. (1996). Where are the gates in gap junction channels. *Clin. Exp. Pharmacol. Physiol.* 23 1047–1052 10.1111/j.1440-1681.1996.tb01167.x 8977158

[B46] Davidson J. S. Baumgarten I. M. (1988). Glycyrrhetinic acid derivatives: a novel class of inhibitors of gap junctional intercellular communication; structure activity relationship. *J. Pharmacol. Exp. Ther.* 246 1104–1107 3418512

[B47] Dean J. B. Ballantyne D. Cardone D. L. Erlichman J. S. Solomon I. C. (2002). Role of gap junctions in CO_2_ chemoreception and respiratory control. *Am. J. Physiol.* 283 665–670 10.1152/ajplung.00142.2002 12225940

[B48] Dean J. B. Huang R. Q. Erlichman J. S. Southard T. L. Hellard D. T. (1997). Cell–cell coupling occurs in dorsal medullary neurons after minimizing anatomicalcoupling artifacts. *Neuroscience* 80 21–40 10.1016/S0306-4522(97)00016-X 9252218

[B49] Dean J. B. Kinkade E. A. Putnam R. W. (2001). Cell–cell coupling in CO_2_/H^+^-excited neurons in brainstem slices. *Respir. Physiol.* 129 83–100 10.1016/S0034-5687(01)00284-5 11738648

[B50] de Carvalho D. Bicego K. C. de Castro O. W. da Silva G. S. Garcia-Cairasco N. Gargaglioni L. H. (2010). Role of neurokinin-1 expressing neurons in the locus coeruleus on ventilatory and cardiovascular responses to hypercapnia. *Respir. Physiol. Neurobiol.* 172 24–31 10.1016/j.resp.2010.04.016 20416403

[B51] Dermietzel R. Spray D. C. (1993). Gap junctions in the brain: where, what type, how many and why? *Trends Neurosci.* 16 186–192 10.1016/0166-2236(93)90151-B 7685944

[B52] Dermietzel R. Traub O. Hwang T. K. Beyer E. Bennet V. L. Spray D. C. (1989). Differential expression of three gap junction proteins in developmental and mature brain tissues. *Proc. Natl. Acad. Sci. U.S.A.* 86 10148–10152 10.1073/pnas.86.24.10148 2557621 PMC298664

[B53] de Souza Moreno V. Bícego K. C. Szawka R. E. Anselmo-Franci J. A. Gargaglioni L. H. (2010). Serotonergic mechanisms on breathing modulation in the rat locus coeruleus. *Pflügers Arch.* 459 357–368 10.1007/s00424-009-0741-4 19844739

[B54] Dillon G. H. Welsh D. E. Waldrop T. G. (1991). Modulation of respiratory reflexes by an excitatory amino acid mechanism in the ventrolateral medulla. *Respir. Physiol.* 85 55–72 10.1016/0034-5687(91)90006-5 1658900

[B55] Edwards F. A. Gibb A. J. (1993). ATP – a fast neurotransmitter. *FEBS Lett.* 325 86–89 10.1016/0014-5793(93)81419-Z 7685715

[B56] Ennis M. Aston-Jones G. (1987). Two physiologically distinct populations of neurons in the ventrolateral medulla innervate the locus coeruleus. *Brain Res.* 425 275–282 10.1016/0006-8993(87)90510-5 3427430

[B57] Ennis M. Aston-Jones G. Shiekhattar R. (1992). Activation of locus coeruleus neurons by nucleus paragigan to cellularis or noxious sensory stimulation is mediated by intracoerulear excitatory amino acid neurotransmission. *Brain Res.* 598 185–195 10.1016/0006-8993(92)90182-9 1336704

[B58] Fabris G. Anselmo-Franci J. A. Branco L. G. (1999). Role of nitric oxide in hypoxia-induced hyperventilation and hypothermia: participation of the locus coeruleus. *Braz. J. Med. Biol. Res.* 32 1389–1398 10.1590/S0100-879X1999001100009 10559840

[B59] Ferreira C. M. de Paula P. M. Branco L. G. (2004). Role of L-glutamate in the locus coeruleus of rats in hypoxia-induced hyperventilation and anapyrexia. *Respir. Physiol. Neurobiol.* 139 157–166 10.1016/j.resp.2003.10.001 15122999

[B60] Filosa J. A. Dean J. B. Putnam R. W. (2002). Role of intracellular and extracellular pH in the chemosensitive response of rat Locus coeruleus neurones. *J. Physiol.* 541(Pt 2) 493–509 10.1113/jphysiol.2001.014142 12042354 PMC2290328

[B61] Fitzgerald P. J. (2012). The NMDA receptor may participate in widespread suppression of circuit level neural activity, in addition to a similarly prominent role in circuit level activation. *Behav. Brain Res.* 230 291–298 10.1016/j.bbr.2012.01.057 22342923

[B62] Foote S. L. Bloom F. E. Aston-Jones G. (1983). Nucleus locus ceruleus: new evidence of anatomical and physiological specificity. *Physiol. Rev.* 63 844–914 6308694 10.1152/physrev.1983.63.3.844

[B63] Fredholm B. B. Abbracchio M. P. Burnstock G. Daly J. W. Harden T. K. Jacobson K. A. (1994). Nomenclature and classification of purinoceptors. *Pharmacol. Rev.* 46 143–156 7938164 PMC4976594

[B64] Frisch C. De Souza-Silva M. A. Söhl G. Güldenagel M. Willecke K. Huston J. P. (2005). Stimulus complexity dependent memory impairment and changes in motor performance after deletion of the neuronal gap junction protein connexin36 in mice. *Behav. Brain Res.* 157 177–185 10.1016/j.bbr.2004.06.023 15617784

[B65] Fukui M. Takishita A. Zhang N. Nakagawa T. Minami M. Satoh M. (2004). Involvement of locus coeruleus noradrenergic neurons in supraspinal antinociceptin by α,β-methylene-ATP in rats. *J. Pharmacol. Sci.* 94 153–160 10.1254/jphs.94.153 14978353

[B66] Gargaglioni L. H. Hartzler L. K. Putnam R. W. (2010). The Locus coeruleus and central chemosensitivity. *Respir. Physiol. Neurobiol.* 173 264–273 10.1016/j.resp.2010.04.024 20435170 PMC2929404

[B67] González A. Smeets W. J. (1993). Noradrenaline in the brain of the South African clawed frog *Xenopus laevis*: a study with antibodies against noradrenaline and dopamine-β-hydroxylase. *J. Comp. Neurol.* 331 363–374 10.1002/cne.903310306 8514914

[B68] Gonzalez M. M. Aston-Jones G. (2006). Circadian regulation of arousal: role of the noradrenergic locus coeruleus system and light exposure. *Sleep* 29 1327–1336 17068987 10.1093/sleep/29.10.1327

[B69] Goodenough D. A. Goliger J. A. Paul D. L. (1996). Connexins, connexons, and intercellular communication. *Annu. Rev. Biochem.* 65 475–502 10.1146/annurev.bi.65.070196.002355 8811187

[B70] Gourine A. V. Melenchuk E. V. Poputnikov D. M. Gourine V. N. Spyer K. M. (2002). Involvement of purinergic signalling in central mechanisms of body temperature regulation in rats. *Br. J. Pharmacol.* 135 2047–2055 10.1038/sj.bjp.0704679 11959809 PMC1573334

[B71] Gourine A. V. Atkinson L. Deuchars J. Spyer K. M. (2003). Purinergic signalling in the medullary mechanisms of respiratory control in the rat: respiratory neurones express the P2X2 receptor subunit. *J. Physiol.* 552(Pt 1) 197–211 10.1113/jphysiol.2003.045294 12878756 PMC2343330

[B72] Gourine A. V. Llaudet E. Dale N. Spyer K. M. (2005). ATP is a mediator of chemosensory transduction in the central nervous system. *Nature* 436 108–111 10.1038/nature03690 16001070

[B73] Haddjeri N. Montigny C. Blier P. (1997). Modulation of the firing activity of noradrenergic neurones in the rat locus coeruleus by the 5-hydroxtryptamine system. *Br. J. Pharmacol.* 120 865–875 10.1038/sj.bjp.0700968 9138693 PMC1564533

[B74] Harms L. Finta E. P. Tschöpl L. M. Illes P. (1992). Depolarization of rat locus coeruleus neurons by adenosine 5^′^-triphosphate. *Neuroscience* 48 941–952 10.1016/0306-4522(92)90282-7 1630630

[B75] Hartfield E. M. Rinaldi F. Glover C. P. Wong L. F. Caldwell M. A. Uney J. B. (2011). Connexin 36 expression regulates neuronal differentiation from neural progenitor cells. *PLoS ONE* 6:e14746 10.1371/journal.pone.0014746 PMC3052311 21408068

[B76] Hartzler L. K. Dean J. B. Putnam R. W. (2007). Developmental changes in the chemosensitive response in locus coeruleus neurons from neonatal rats. *Soc. Neurosci. Abstr.* 33 297–298

[B77] Hayashida K. Parker R. A. Eisenach J. C. (2010). Activation of glutamate transporters in the locus coeruleus paradoxically activates descending inhibition in rats. *Brain Res.* 1317 80–86 10.1016/j.brainres.2009.12.086 20059984 PMC2822016

[B78] Henny P. Brischoux F. Mainville L. Stroh T. Jones B. E. (2010). Immunohistochemical evidence for synaptic release of glutamate from orexin terminals in the locus coeruleus. *Neuroscience* 169 1150–1157 10.1016/j.neuroscience.2010.06.003 20540992 PMC2914109

[B79] Hermann D. M. Luppi P. H. Peyron C. Hinckel P. Jouvet M. (1997). Afferent projections to the rat nuclei raphe magnus, raphe pallidus and reticularis gigantocellularis pars α demonstrated by iontophoretic application of choleratoxin (subunit b). *J. Chem. Neuroanat.* 13 1–21 10.1016/S0891-0618(97)00019-7 9271192

[B80] Hewitt A. Barrie R. Graham M. Bogus K. Leiter J. C. Erlichman J. S. (2004). Ventilatory effects of gap junction blockade in the RTN in awake rats. *Am. J. Physiol. Regul. Integr. Comp. Physiol.* 287 1407–1418 10.1152/ajpregu.00404.2004 15308490

[B81] Hilaire G. Viemari J. C. Coulon P. Simonneau M. Bévengut M. (2004). Modulation of the respiratory rhythm generator by the pontine noradrenergic A5 and A6 groups in rodents. *Respir. Physiol. Neurobiol.* 143 187–197 10.1016/j.resp.2004.04.016 15519555

[B82] Holloway B. B. Stornetta R. L. Bochorishvili G. Erisir A. Viar K. E. Guyenet P. (2013). Monosynaptic glutamatergic activation of locus coeruleus and other lower brainstem noradrenergic neurons by the C1 cells in mice. *J. Neurosci.* 33 18792–18805 10.1523/JNEUROSCI.2916-13.2013 24285886 PMC3841449

[B83] Homayoun H. Moghaddam B. (2007). NMDA receptor hypofunction produces opposite effects on prefrontal cortex interneurons and pyramidal neurons. *J. Neurosci.* 27 11496–11500 10.1523/JNEUROSCI.2213-07.2007 17959792 PMC2954603

[B84] Hooper M. L. Subak-Sharpe J. H. (1981). Metabolic co-operation between cells. *Int. Rev. Cytol.* 69 45–104 6260700 10.1016/s0074-7696(08)62320-7

[B85] Horvath T. L. Diano S. Van den Pol A. N. (1999). Synaptic interaction between hypocretin (orexin) and neuropeptide Y cells in the rodent and primate hypothalamus: a novel circuit implicated in metabolic and endocrine regulations. *J. Neurosci.* 19 1072–1087 9920670 10.1523/JNEUROSCI.19-03-01072.1999 PMC6782143

[B86] Huxtable A. G. Zwicker J. D. Poon B. Y. Pagliardini S. Vrouwe S. Q. Greer J. J. (2009). Tripartite purinergic modulation of central respiratory networks during perinatal development: the influence of ATP, ectonucleotidases, and ATP metabolites. *J. Neurosci.* 29 14713–14725 10.1523/JNEUROSCI.2660-09.2009 19940166 PMC6666021

[B87] Iijima K. Ohtomo K. (1988). Immunocytochemical study using a GABA antiserum for the demonstration of inhibitory neurons in the rat locus ceruleus. *Am. J. Anat.* 181 43–52 10.1002/aja.1001810106 3348147

[B88] Illes P. Sevcik J. Finta E. P. Frohlich R. Nieber K. Norenberg W. (1994). Modulation of locus coeruleus neurons by extra- and intracellular adenosine 50-triphosphate. *Brain Res. Bull.* 35 513–519 10.1016/0361-9230(94)90165-1 7859109

[B89] Ishimatsu M. Williams J. T. (1996). Synchronous activity in locus coeruleus results from dendritic interactions in pericoerulear regions. *J. Neurosci.* 16 5196–5204 8756448 10.1523/JNEUROSCI.16-16-05196.1996 PMC6579296

[B90] Jacobs B. L. Azmitia E. C. (1992). Structure and function of the brain serotonin system. *Physiol. Rev.* 72 165–229 1731370 10.1152/physrev.1992.72.1.165

[B91] Jones B. E. (2005). “Basic mechanisms of sleep-wake states,” in *Principles and Practice of Sleep Medicine*, eds Kryger M. H. Roth T. Dement W. C. (Philadelphia:Elsevier Saunders) 136–153 10.1016/B0-72-160797-7/50018-5

[B92] Jones B. E. Yang T. Z. (1985). The efferent projections from the reticular formation and the locus coeruleus studied by anterograde and retrograde axonal transport in the rat. *J. Comp. Neurol.* 242 56–92 10.1002/cne.902420105 2416786

[B93] Jouvet M. (1999). Sleep and serotonin: an unfinished story. *Neuropsychopharmacology* 21(Suppl. 1) 24S–27S 10.1016/S0893-133X(99)00009-3 10432485

[B94] Kaehler S. T. Singewald N. Philippu A. (1999). Dependence of serotonin release in the locus coeruleus on dorsal raphe neuronal activity. *Naunyn Schmiedebergs Arch. Pharmacol.* 359 386–393 10.1007/PL00005365 10498288

[B95] Kanjhan R. Housley G. D. Burton L. D. Christie D. L. Kippenberger A. Thorne P. R. (1999). Distribution of the P2X2 receptor subunit of the ATP-gated ion channels in the rat central nervous system. *J. Comp. Neurol.* 407 11–32 10.1002/(SICI)1096-9861(19990428)407:1<11::AID-CNE2>3.0.CO;2-R 10213185

[B96] Khakpay R. Polster D. Köles L. Skorinkin A. Szabo B. Wirkner K. (2010). Potentiation of the glutamatergic synaptic input to rat locus coeruleus neurons by P2X7 receptors. *Purinergic Signal.* 6 349–359 10.1007/s11302-010-9198-3 21103218 PMC2947656

[B97] Kim M. A. Lee H. S. Lee B. Y. Waterhouse B. D. (2004). Reciprocal connections between subdivisions of the dorsal raphe and the nuclear core of the locus coeruleus in the rat. *Brain Res.* 1026 56–67 10.1016/j.brainres.2004.08.022 15476697

[B98] Kumar N. M. Gilula N. B. (1996). The gap junction communication channel. *Cell* 84 381–388 10.1016/S0092-8674(00)81282-9 8608591

[B99] Kuwahata T. (2004). Effects of adenosine and ATP on the membrane potential and synaptic transmission in neurons of the rat locus coeruleus. *Kurume Med. J.* 51 109–123 10.2739/kurumemedj.51.109 15373228

[B100] Laird D. W. (2006). Life cycle of connexins in health and disease. *Biochem. J.* 394 527–543 10.1042/BJ20051922 16492141 PMC1383703

[B101] Li Z. Y. Xia B. L. Huang C. J. (1992). Effects of microinjection of L-glutamate into locus coeruleus complex area on respiration. *J. Tongji Med. Univ.* 12 205–208 10.1007/BF02887850 1289566

[B102] Liu G. Feldman J. L. Smith J. C. (1990). Excitatory amino acid-mediated transmission of inspiratory drive to phrenic motoneurons. *J. Neurophysiol.* 64 423–436 1976765 10.1152/jn.1990.64.2.423

[B103] Llewellyn-Smith I. J. Burnstock G. (1998). Ultrastructural localization of P2X3 receptors in rat sensory neurons. *Neuroreport* 9 2545–2550 10.1097/00001756-199808030-00022 9721930

[B104] Loewenstein W. R. (1981). Junctional intercellular communication: the cell-to-cell membrane channel. *Physiol. Rev.* 61 829–913 6270711 10.1152/physrev.1981.61.4.829

[B105] Lorier A. R. Huxtable A. G. Robinson D. M. Lipski J. Housley G. D. Funk G. D. (2007). P2Y1 receptor modulation of the pre-Bötzinger complex inspiratory rhythm generating network *in vitro*. *J. Neurosci.* 27 993–1005 10.1523/JNEUROSCI.3948-06.2007 17267553 PMC6673186

[B106] Lorier A. R. Lipski J. Housley G. D. Greer J. J. Funk G. D. (2008). ATP sensitivity of preBötzinger complex neurones in neonatal rat *in vitro*: mechanism underlying a P2 receptor-mediated increase in inspiratory frequency. *J. Physiol.* 586 1429–1446 10.1113/jphysiol.2007.143024 18174215 PMC2375674

[B107] Luppi P. H. Aston-Jones G. Akaoka H. Chouvet G. Jouvet M. (1995). Afferent projections to the rat locus coeruleus demonstrated by retrograde and anterograde tracing with cholera-toxin B subunit and PHA-L. *Neuroscience* 65 119–160 10.1016/0306-4522(94)00481-J 7753394

[B108] Mateo J. García-Lecea M. Miras-Portugal M. T. Castro E. (1998). Ca^2+^ signals mediated by P2X-type purinoceptors in cultured cerebellar Purkinje cells *J. Neurosci.* 18 1704–1712 9464995 10.1523/JNEUROSCI.18-05-01704.1998 PMC6792619

[B109] Meltzer H. Y. Lowy M. T. (1987). “The serotonin hypothesis of depression,” in *Psychopharmacology: The Third Generation of Progress* ed. Meltzer H. Y. (New York, NY:Raven Press) 513–525

[B110] Miyawaki T. Kawamura H. Komatsu K. Yasugi T. (1991). Chemical stimulation of the locus coeruleus: inhibitory effects on hemodynamics and renal sympathetic nerve activity. *Brain Res.* 568 101–108 10.1016/0006-8993(91)91384-D 1687667

[B111] Mokha S. S. McMillan J. A. Iggo A. (1985). Descending control of spinal nociceptive transmission. Actions produced on spinal multireceptive neurones from the nuclei locus coeruleus (LC) and raphe magnus (NRM). *Exp. Brain. Res.* 58 213–226 10.1007/BF00235305 2987012

[B112] Morgane P. J. Jacobs M. S. (1979). Raphe projections to the locus coeruleus in the rat. *Brain Res. Bull.* 4 519–534 10.1016/0361-9230(79)90037-6 226233

[B113] Nadarajah B. Jones A. M. Evans W. H. Parnavelas J. G. (1997). Differential expression of connexins during neocortical development and neuronal circuit formation. *J. Neurosci.* 17 3096–3111 9096144 10.1523/JNEUROSCI.17-09-03096.1997 PMC6573667

[B114] Nattie E. (1999). CO_2_, brainstem chemoreceptors and breathing. *Prog. Neurobiol.* 59 299–331 10.1016/S0301-0082(99)00008-8 10501632

[B115] Naus C. C. G. Bani-Yaghoub M. (1998). Gap junctional communication in the developing central nervous system. *Cell Biol. Int.* 22 751–763 10.1006/cbir.1998.0391 10873289

[B116] Nestler E. J. Alreja M. Aghajanian G. K. (1999). Molecular control of locus coeruleus neurotransmission. *Biol. Psychiatry* 46 1131–1139 10.1016/S0006-3223(99)00158-4 10560020

[B117] Nichols N. L. Hartzler L. K. Conrad S. C. Dean J. B. Putnam R.W. (2008). Intrinsic chemosensitivity of individual nucleus tractus solitarius (NTS) and locus coeruleus (LC) neurons from neonatal rats. *Adv. Exp. Biol. Med.* 605 348–352 10.1007/978-0-387-73693-8_61 18085298

[B118] Nieber K. Poelchen W. Illes P. (1997). Role of ATP in fast excitatory synaptic potentials in locus coeruleus neurones of the rat. *Br. J. Pharmacol.* 122 423–430 10.1038/sj.bjp.0701386 9351497 PMC1564950

[B119] Noronha-de-Souza C. R. Bícego K. C. Michel G. Glass M. L. Branco L. G. S. Gargaglioni L. H. (2006). Locus coeruleus is a central chemoreceptive site in toads. *Am. J. Physiol. Regul. Integr. Comp. Physiol.* 291 997–1006 10.1152/ajpregu.00090.2006 16644910

[B120] Nygren L. G. Olson L. (1977). A new major projection from locus coeruleus: the main source of noradrenergic nerve terminals in the ventral and dorsal columns of the spinal cord. *Brain Res.* 132 85–93 10.1016/0006-8993(77)90707-7 890479

[B121] Olpe H. R. Steinmann M. (1991). Responses of locus coeruleus neurons to neuropeptides. *Prog. Brain Res.* 88 241–248 10.1016/S0079-6123(08)63813-3 1813923

[B122] Oyamada Y. Andrzejewski M. Mückenhoff K. Scheid P. Ballantyne D. (1999). Locus coeruleus neurones *in vitro*: pH-sensitive oscillations of membrane potential in an electrically coupled network. *Respir. Physiol.* 118(2–3), 131–147 10.1016/S0034-5687(99)00088-2 10647858

[B123] Oyamada Y. Ballantyne D. Muckenhoff K. Scheid P. (1998). Respiration-modulated membrane potential and chemosensitivity of locus coeruleus neurones in the *in vitro* brainstem–spinal cord of the neonatal rat. *J. Physiol.* 513(Pt 2) 381–398 10.1111/j.1469-7793.1998.381bb.x 9806990 PMC2231289

[B124] Parisian K. Wages P. Smith A. Jarosz J. Hewitt A. Leiter J. C. (2004). Ventilatory effects of gap junction blockade in the NTS in awake rats. *Respir. Physiol. Neurobiol.* 142 127–143 10.1016/j.resp.2004.06.014 15450475

[B125] Pasquier D. A. Kemper T. L. Forbes W. B. Morgane P. J. (1977). Dorsal raphe, substantia nigra and locus coeruleus: interconnections with each other and the neostriatum. *Brain Res. Bull.* 2 323–339 10.1016/0361-9230(77)90066-1 922511

[B126] Patrone L. G. A. Bícego K. C. Hartzler L. K. Putnam R. W. Gargaglioni L. H. (2014). Cardiorespiratory effects of gap junction blockade in the locus coeruleus in unanesthetized adult rats. *Respir. Physiol. Neurobiol.* 190 86–95 10.1016/j.resp.2013.09.001 24035835

[B127] Petralia R. S. Yokotani N. Wenthold R. J. (1994). Light and electron microscope distribution of the NMDA receptor subunit NMDAR1 in the rat nervous system using a selective anti-peptide antibody. *J. Neurosci.* 14 667–696 8301357 10.1523/JNEUROSCI.14-02-00667.1994 PMC6576818

[B128] Peyron C. Luppi P. H. Fort P. Rampon C. Jouvet M. (1996). Lower brainstem catecholamine afferents to the rat dorsal raphe nucleus. *J. Comp. Neurol.* 364 402–413 10.1002/(SICI)1096-9861(19960115)364:3<402::AID-CNE2>3.0.CO;2-8 8820873

[B129] Peyron C. Tighe D. K. van den Pol A. N. de Lecea L. Heller H. C. Sutcliffe J. G. (1998). Neurons containing hypocretin (orexin) project to multiple neuronal systems. *J. Neurosci.* 18 9996–10015 9822755 10.1523/JNEUROSCI.18-23-09996.1998 PMC6793310

[B130] Pickel V. Joh T. H. Reis D. J. (1977) A serotoninergic innervation of noradrenergic neurons in nucleus locus coeruleus: demonstration by immunocytochemical localization of the transmitter specific enzyme tyrosine and tryptophan hydroxylase. *Brain Res.* 131 197–214 10.1016/0006-8993(77)90515-7 19125

[B131] Picoli C. Nouvel V. Aubry F. Reboul M. Duchêne A. Jeanson T. (2012). Human connexin channel specificity of classical and new gap junction inhibitors. *J. Biomol. Screen.* 17 1339–1347 10.1177/1087057112452594 22786894

[B132] Pierce M. L. Deuchars J. Deuchars S. A. (2010). Spontaneous rhythmogenic capabilities of sympathetic neuronal assemblies in the rat spinal cord slice. *Neuroscience* 170 827–838 10.1016/j.neuroscience.2010.07.007 20650307 PMC2989444

[B133] Pieribone V. A. Aston-Jones G. (1991). Adrenergic innervation of the rat nucleus locus coeruleus arises predominantly from the C1 adrenergic cell group in the rostral medulla. *Neuroscience* 41 525–542 10.1016/0306-4522(91)90346-P 1714551

[B134] Pineda J. Aghajanian G. K. (1997). Carbon dioxide regulates the tonic activity of locus ceruleus neurons by modulating a proton- and polyamine-sensitive inward rectifier potassium current. *Neuroscience* 77 723–743 10.1016/S0306-4522(96)00485-X 9070748

[B135] Poelchen W. Sieler D. Wirkner K. Illes P. (2001). Co-transmitter function of ATP in central catecholaminergic neurons of the rat. *Neuroscience* 102 593–602 10.1016/S0306-4522(00)00529-7 11226696

[B136] Pollard H. Llorens-Cortes C. Barbin G. Garbarg M. Schwartz J. C. (1978). Histamine and histidine decarboxylase in brain stem nuclei: distribution and decrease after lesions. *Brain Res.* 157 178–181 10.1016/0006-8993(78)91010-7 698844

[B137] Putnam R. W. (2001). Intracellular pH regulation of neurons in chemosensitive and nonchemosensitive areas of brain slices. *Respir. Physiol.* 129 37–56 10.1016/S0034-5687(01)00281-X 11738645

[B138] Putnam R. W. Filosa J. A. Ritucci N. A. (2004) Cellular mechanisms involved in CO_2_ and acid signaling in chemosensitive neurons. *Am. J. Physiol. Cell. Physiol*. 287 1493–1526 10.1152/ajpcell.00282.2004 15525685

[B139] Rash J. E. Olson C. O. Davidson K. G. Yasumura T. Kamasawa N. Nagy J. I. (2007). Identification of connexin 36 in gap junctions between neurons in rodent locus coeruleus. *Neuroscience* 147 938–956 10.1016/j.neuroscience.2007.04.061 17601673 PMC2034517

[B140] Redmond D. J. Huang Y. H. (1979). Current concepts. II. New evidencefor a locus coeruleus-norepinephrine connection with anxiety. *Life Sci.* 25 2149–2162 10.1016/0024-3205(79)90087-0 120478

[B141] Reil J. C. (1809). Untersuchungen uber den Bau des grossen Gehims im Menschen. *Arch. Physiol.* 9 136–524

[B142] Rekling J. C. Feldman J. L. (1997). Bidirectional electrical coupling between inspiratory motoneurons in the newborn mouse nucleus ambiguus. *J. Neurophysiol.* 78 3508–3510 9405571 10.1152/jn.1997.78.6.3508

[B143] Ruggiero R. N. Bueno-Juúnior L. S. Ross J. B. Fachim H. A. Padovan-Neto F. E. Merlo S. (2011). Neurotransmissão glutamateérgica e a plasticidade sinaáptica: aspectos moleculares, clínicos e filogenéticos. *Medicina (Ribeirão Preto)* 44 143–156 10.11606/issn.2176-7262.v44i2p143-156

[B144] Russell G. V. (1955). The nucleus locus coeruleus (dorsolateralis tegmenti). *Tex. Rep. Biol. Med.* 13 939–988 13281797

[B145] Samuels E.R. Szabadi E. (2008). Functional neuroanatomy of the noradrenergic locus coeruleus: its roles in the regulation of arousal and autonomic function part Il: Physiological and Pharmacological Manipulations and Pathological Alterations of Locus Coeruleus Activity in Humans. *Curr. Neuropharmacol.* 6(3) 254–285 10.2174/157015908785777193 19506724 PMC2687931

[B146] Santin J. M. Hartzler L. K. (2013). Respiratory signaling of locus coeruleus neurons during hypercapnic acidosis in the bullfrog, *Lithobates catesbeianus*. *Respir. Physiol. Neurobiol.* 185 553–561 10.1016/j.resp.2012.11.002 23146875

[B147] Saper C. B. Chou T. C. Scammell T. E. (2001). The sleep switch: hypothalamic control of sleep and wakefulness. *Trends Neurosci.* 24 726–731 10.1016/S0166-2236(00)02002-6 11718878

[B148] Sato K. Kiyama H. Tohyama M. (1993). The differential expression patterns of messenger RNAs encoding non-*N*-methyl-D-aspartate glutamate receptor subunits (GluR1-4) in the rat brain. *Neuroscience* 52 515–539 10.1016/0306-4522(93)90403-3 8450957

[B149] Scheibler P. Pesic M. Franke H. Reinhardt R. Wirkner K. Illes P. (2004). P2X2 and P2Y1 immunofluorescence in rat neostriatal medium-spiny projection neurones and cholinergic interneurones is not linked to respective purinergic receptor function. *Br. J. Pharmacol.* 143 119–131 10.1038/sj.bjp.0705916 15345659 PMC1575277

[B150] Schreihofer A. M. Stornetta R. L. Guyenet P. G. (1999). Evidence for glycinergic respiratory neurons: Botzinger neurons express mRNA for glycinergic transporter 2. *J. Comp. Neurol.* 407 583–597 10.1002/(SICI)1096-9861(19990517)407:4<583::AID-CNE8>3.0.CO;2-E 10235646

[B151] Segal M. (1979). Serotonergic innervation of the locus coeruleus from the dorsal raphe and its action on responses to noxious stimuli. *J. Physiol.* 286 401–415 439032 10.1113/jphysiol.1979.sp012628 PMC1281580

[B152] Seiver L. J. (1987). “Role of noradrenergic mechanisms in the etiology of the affective disorders,” in *Psychopharmacology: The Third Generation of Progress* ed. Meltzer H. Y. (New York, NY:Raven Press) 493–504

[B153] Shen K. Z. North R. A. (1993). Excitation of rat locus coeruleus neurons by adenosine 5^′^-triphosphate: ionic mechanism and receptor characterization. *J. Neurosci.* 13 894–899 8441014 10.1523/JNEUROSCI.13-03-00894.1993 PMC6576587

[B154] Singewald N. Philippu A. (1998). Release of neurotransmitters in the locus coeruleus. *Prog. Neurobiol.* 56 237–267 10.1016/S0301-0082(98)00039-2 9760703

[B155] Sohl G. Degen J. Teubner B. Willecke K. (1998). The murine gap junction gene connexin36 is highly expressed in mouse retina and regulated during brain development. *FEBS Lett.* 428 27–31 10.1016/S0014-5793(98)00479-7 9645468

[B156] Solomon I. C. (2003). Connexin 36 distribution in putative CO_2_-chemosensitive brainstem regions in rat. *Respir. Physiol. Neurobiol.* 139 1–20 10.1016/j.resp.2003.09.004 14637306

[B157] Solomon I. C. Dean J. B. (2002). Gap junctions in CO_2_-chemoreception and respiratory control. *Respir. Physiol. Neurobiol.* 131 155–173 10.1016/S1569-9048(02)00090-3 12126918

[B158] Solomon I. C. Edelman N. H. Neubauer J. A. (2000). Pre-Bötzinger complex functions as a central hypoxia chemosensor for respiration *in vivo.* *J. Neurophysiol.* 83 2854–2868 10805683 10.1152/jn.2000.83.5.2854

[B159] Solomon I. C. Halat T. J. El-Maghrabi M. R. O’Neal M. H. (2001). Localization of connexin 26 and connexin 32 in putative CO_2_-chemosensitivie brainstem regions in rat. *Respir. Physiol.* 129 101–121 10.1016/S0034-5687(01)00299-7 11738649

[B160] Spyer K. M. Dale N. Gourine A. V. (2004). ATP is a key mediator of central and peripheral chemosensory transduction. *Exp. Physiol.* 98 53–59 10.1113/expphysiol.2003.002659 15109209

[B161] Sutin E. L. Jacobowitz D. M. (1991). Neurochemicals in the dorsal pontine tegmentum. *Prog. Brain Res.* 88 3–14 10.1016/S0079-6123(08)63796-6 1726029

[B162] Sved A. F. Felsten G. (1987). Stimulation of the locus coeruleus decreases arterial pressure. *Brain Res.* 414 119–132 10.1016/0006-8993(87)91332-1 2887237

[B163] Swanson L. W. Hartman B. K. (1975). The central adrenergic system. An immunofluorescence study of the location of cell bodies and their efferent connections in the rat utilizing dopamine-β-hydroxylase as a marker. *J. Comp. Neurol.* 163 467–505 10.1002/cne.901630406 1100685

[B164] Swanson L. W. Sawchenko P. E. (1983). Hypothalamic integration: organization of the paraventricular and supraoptic nuclei. *Annu. Rev. Neurosci.* 6 269–324 10.1146/annurev.ne.06.030183.001413 6132586

[B165] Szabo S. T. Blier P. (2001). Functional and pharmacological characterization of the modulatory role of serotonin on the firing activity of locus coeruleus norepinephrine neurons. *Brain Res.* 922 9–20 10.1016/S0006-8993(01)03121-3 11730697

[B166] Taneja P. Ogier M. Brooks-Harris G. Schmid D. A. Katz D. M. Nelson S. B. (2009). Pathophysiology of locus ceruleus neurons in a mouse model of Rett syndrome. *J. Neurosci.* 29 12187–12195 10.1523/JNEUROSCI.3156-09.2009 19793977 PMC2846656

[B167] Taxini C. L. Puga C. C. I. Dias M. B. Bícego K. C. Gargaglioni L. H. (2013). Ionotropic but not metabotropic glutamatergic receptors in the locus coeruleus modulate the hypercapnic ventilatory response in unanaesthetized rats. *Acta Physiol.* 208 125–135 10.1111/apha.12082 23414221

[B168] Thomas T. Ralevic V. Bardini M. Burnstock G. Spyer K. M. (2001). Evidence for the involvement of purinergic signalling in the control of respiration. *Neuroscience* 107 481–490 10.1016/S0306-4522(01)00363-3 11719002

[B169] Thomas T. Spyer K. M. (2000). ATP as a mediator of mammalian central CO_2_ chemoreception. *J. Physiol.* 523(Pt 2), 441–447 10.1111/j.1469-7793.2000.00441.x 10699087 PMC2269817

[B170] Törk I. (1990). Anatomy of the serotonergic system. *Ann. N. Y. Acad. Sci.* 600 9–34; discussion 34–35 10.1111/j.1749-6632.1990.tb16870.x 2252340

[B171] Travagli R. A. Dunwiddie T. V. Williams J. T. (1995). Opioid inhibition in locus coeruleus. *J. Neurophysiol.* 74 518–528 7472359 10.1152/jn.1995.74.2.519

[B172] Tschöpl M. Harms L. Norenberg W. Illes P. (1992). Excitatory effects of adenosine 5^′^-triphosphate on rat locus coeruleus neurones. *Eur. J. Pharmacol.* 213 71–77 10.1016/0014-2999(92)90234-U 1499658

[B173] Uhde T. W. Boulenger J. P. Post R. M. Siever L. J. Vittone B. J. Jimerson D. C. (1984). Fear and anxiety: relationship to noradrenergic function. *Psychopathology* 17(Suppl. 3) 8–23 10.1159/000284127 6505121

[B174] Van Bockstaele E. J. Pieribone V. A. Aston-Jones G. (1989) Diverse afferents converge on the nucleus paragigantocellularis in the rat ventrolateral medulla: retrograde and anterograde tracing studies. *J. Comp. Neurol.* 290 561–584 10.1002/cne.902900410 2482306

[B175] Vertes R. Kocsis B. (1994) Projections of the dorsal raphe nucleus to the brainstem: PHA-L analysis in the rat. *J. Comp. Neurol.* 340 11–26 10.1002/cne.903400103 8176000

[B176] Viemari J. C. Bévengut M. Burnet H. Coulon P. Pequignot J. M. Tiveron M. C. (2004). Phox2a gene, A6 neurons, and noradrenaline are essential for development of normal respiratory rhythm in mice. *J. Neurosci.* 24 928–937 10.1523/JNEUROSCI.3065-03.2004 14749437 PMC6729821

[B177] Virginio C. MacKenzie A. Rassendren F. A. North R. A. Surprenant A. (1999). Pore dilation of neuronal P2X receptor channels. *Nat. Neurosci.* 2 315–321 10.1038/7225 10204537

[B178] von Kügelgen I. Späth L. Starke K. (1994). Evidence for P2-purinoceptor-mediated inhibition of noradrenaline release in the rat brain cortex. *Br. J. Pharmacol.* 113 815–822 10.1111/j.1476-5381.1994.tb17066.x 7858872 PMC1510427

[B179] von Kügelgen I. Stöffel D. Starke K. (1995). P2-purinoceptor-mediated inhibition of noradrenaline release in rat atria. *Br. J. Pharmacol.* 115 247–254 10.1111/j.1476-5381.1995.tb15870.x 7670726 PMC1908323

[B180] Vulchanova L. Riedl M. S. Shuster S. J. Buell G. Surprenant A. North R. A. (1997). Immunohistochemical study of the P2X2 and P2X3 receptor subunits in rat and monkey sensory neurons and their central terminals. *Neuropharmacology* 36 1229–1242 10.1016/S0028-3908(97)00126-3 9364478

[B181] Westlund K. N. Bowker R. M. Ziegler M. G. Coulter J. D. (1983) Noradrenergic projections to the spinal cord of the rat. *Brain Res.* 263 15–31 10.1016/0006-8993(83)91196-4 6839168

[B182] Williams J. T. Henderson G. North R. A. (1985). Characterization of α 2-adrenoceptors which increase potassium conductance in rat locus coeruleus neurones. *Neuroscience* 14 95–101 10.1016/0306-4522(85)90166-6 2579354

[B183] Williams J. T. Marshall K. C. (1987). Membrane properties and adrenergic responses in locus coeruleus neurones of young rats. *J. Neurosci.* 7 3687–3694 2890723 10.1523/JNEUROSCI.07-11-03687.1987 PMC6569025

[B184] Wisden W. Seeburg P. H. (1993). A complex mosaic of high-affinity kainate receptors in rat brain. *J. Neurosci.* 13 3582–3598 8393486 10.1523/JNEUROSCI.13-08-03582.1993 PMC6576517

[B185] Yao S. T. Barden J. A. Finkelstein D. I. Bennett M. R. Lawrence A. J. (2000). Comparative study on the distribution patterns of P2X1–P2X6 receptor immunoreactivity in the brainstem of the rat and the common marmoset (*Callithrix jacchus*): association with catecholamine cell groups. *J. Comp. Neurol.* 427 485–507 10.1002/1096-9861(20001127)427:4<485::AID-CNE1>3.0.CO;2-S 11056460

[B186] Yao S. T. Lawrence A. J. (2005). Purinergic modulation of cardiovascular function in the rat locus coeruleus. *Br. J. Pharmacol.* 145 342–352 10.1038/sj.bjp.0706179 15735655 PMC1576143

